# Shedding of *Salmonella* Typhimurium in vaccinated and unvaccinated hens during early lay in field conditions: a randomised controlled trial

**DOI:** 10.1186/s12866-018-1201-0

**Published:** 2018-07-20

**Authors:** Pardeep Sharma, Charles Caraguel, Margaret Sexton, Andrea McWhorter, Greg Underwood, Karen Holden, Kapil Chousalkar

**Affiliations:** 1 School of Animal and Veterinary Science, The University of Adelaide, Roseworthy, South Australia 5371 Australia; 2 Biosecurity SA, Primary Industries and Regions SA, Adelaide, SA Australia; 3 Bioproperties, c/RMIT University, Bundoora, VIC 3083 Australia

**Keywords:** *Salmonella* typhimurium, Vaccine, Layer hens, Randomized controlled trial, Early lay

## Abstract

**Background:**

*Salmonella* vaccination is one of the control measure that farmers can use to reduce bacterial shedding in their flocks. This study aimed to examine the efficacy of the Vaxsafe® ST (Strain STM-1) attenuated live vaccine administered as ocular and oral doses followed by an intramuscular (IM) dose in rearing, in reducing contamination by *Salmonellae* of both eggs and the environment in the commercial multi-age cage layer sheds. A randomised controlled trial was conducted up to 26 weeks post last vaccine on two different multi-age caged egg farms.

**Results:**

No clinical symptoms were observed following IM administration of STM-1 during rearing. Following the first two STM-1 doses, both vaccinated and unvaccinated birds exhibited antibody titres below the positive cut-off value, however after IM administration of STM-1, antibody titres in the vaccinated group were above the cut-off value. Wild type *Salmonella* Typhimurium was not detected during the rearing of pullets. During production, the antibody titres were significantly higher in the vaccinated group at all sampling points during this trial. There was no significant difference in the prevalence of *Salmonella* (detected by culture and PCR method) between the vaccinated and unvaccinated groups on the egg belt and faeces in early lay. Wild-type *Salmonella* spp. were consistently found in dust samples. Quantitative PCR (qPCR) assay was able to differentiate between the live vaccine strain and wild type *Salmonella*. The load of wild-type *Salmonella* in shed environment was relatively low (1.3 log_10_ ± 0.48 CFU/m^2^ of surface area).

**Conclusion:**

Given that *Salmonella* Typhimurium and other serovars are able to survive/persist in the shed environment (such as in dust), regular cleaning and or removal of dust from shed is important. Use of the Vaxsafe® ST vaccine in multi-age flocks is “not an ultimate intervention” for reduction of *Salmonella* Typhimurium because of the complexities involved in achieving control, such as the efficacy of cleaning of sheds, the lack of resting periods between batches and the possible carry over of contamination from existing flocks. Hence implementation of more than one or several interventions strategies is essential.

**Electronic supplementary material:**

The online version of this article (10.1186/s12866-018-1201-0) contains supplementary material, which is available to authorized users.

## Background

*Salmonella* vaccination is one practical measure farmers can use to reduce bacterial shedding in their flocks [1, 2]. Vaccination confers immunological protection against infection to layer hens and reduces on-farm contamination [3–5]. Both live and killed *Salmonella* vaccines have been used with variable success in laying hens [6]. I Gantois, R Ducatelle, L Timbermont, F Boyen, L Bohez, F Haesebrouck, F Pasmans and F van Immerseel [7] tested a live metabolic drift mutant vaccine TAD *Salmonella* vac® E and TAD *Salmonella* vac® T against *Salmonella* Enteritidis (SE) challenge in laying hens and found that vaccination reduced bacterial colonisation of the reproductive organs and intestinal tracts, ultimately reducing egg contamination. *Salmonella* Typhimurium (ST) is a major serovar in the Australian egg industry, yet there is a lack of vaccine efficacy data in laying hens. Vaxsafe® ST (Bioproperties Pty Ltd., Australia) is the only live attenuated vaccine registered for the control of ST infection in poultry in Australia. Vaxsafe® ST (STM-1) was developed for short-lived birds (such as broilers) and registered for oral and coarse-spray application routes. STM-1 was engineered from a virulent wild-type *S*. Typhimurium by disrupting the *aroA* gene using a transposon (aroA-554: Tn 10) insertion method [8]. While studies have been conducted to test the efficacy of a range of different *Salmonella* vaccines in chickens under experimental conditions [9–13], there is limited information on the efficiency of STM-1 in hens challenged naturally under field conditions. The primary aim of this trial was to investigate the efficacy of STM-1 in commercial egg laying flocks, naturally infected with *S.* Typhimurium during early lay. Furthermore, two live vaccinations (oral) followed by parenteral administration (IM injection) prior to the onset of egg production, has not been evaluated in randomized controlled trials.

## Results

### Effects of STM-1 vaccination on pullets during rearing

All three rearing sheds (A, B and C) were *Salmonella* negative prior to the arrival of the chicks. Chick meconium samples collected before administration of Vaxsafe® ST were also *Salmonella* negative. No clinical symptoms were observed following IM administration of STM-1.

**Fig. 1:**
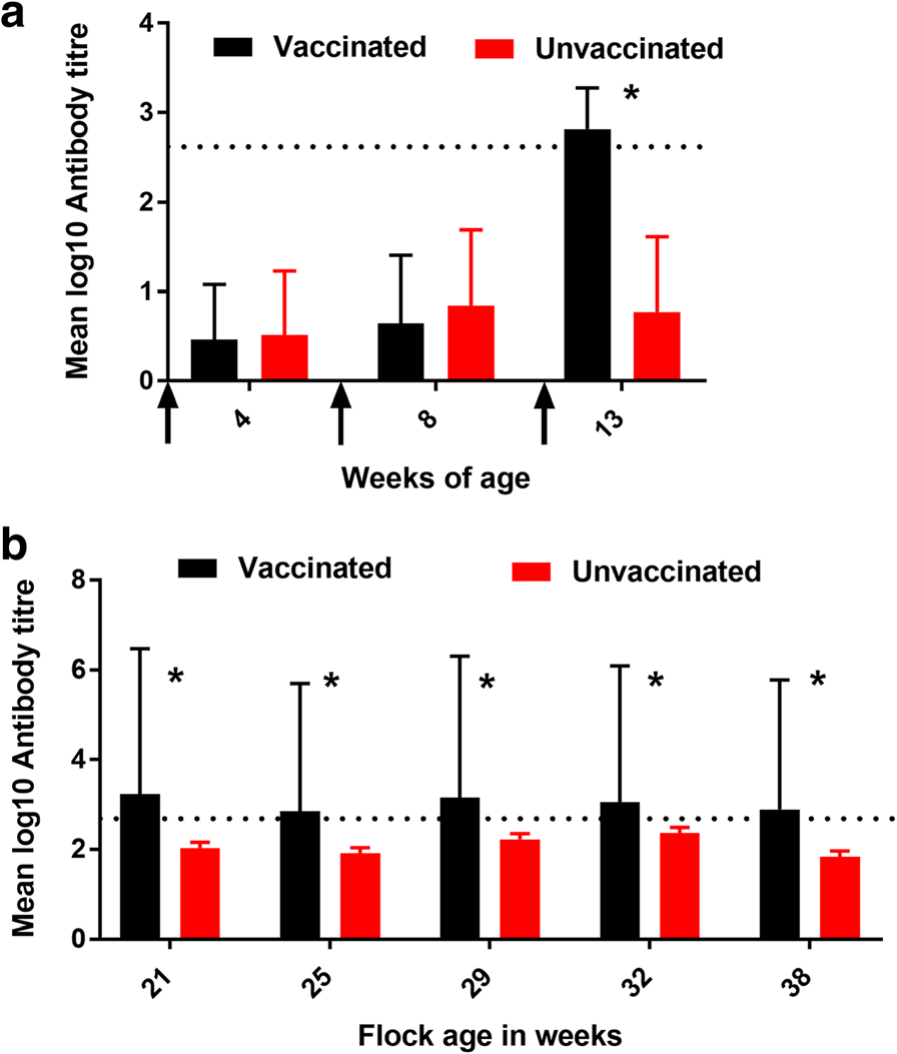
**a** Antibody titers in vaccinated and unvaccinated pullets during rearing. Arrows indicate the timing of vaccine administration. At each sampling point, blood (n = 15 each from vaccinated and unvaccinated groups) from jugular vein was collected. Data was analysed by ANOVA. No significant differences were detected between vaccinated and unvaccinated group at week 4 and 8. At week 13, antibody titers in vaccinated flock was significantly higher (p < 0.01) compared to unvaccinated flock (**b**) Antibody titers in vaccinated and unvaccinated hens during early lay. At each sampling point, blood (n = 10 each from vaccinated and unvaccinated groups) from jugular vein was collected. Data was analysed by ANOVA. Antibody titers in vaccinated flock were significantly higher (p < 0.0001) compared to unvaccinated flock

Following the first two STM-1 doses, both vaccinated and unvaccinated birds exhibited antibody titres below the positive cut-off value. Following IM administration of STM-1, antibody titres in the vaccinated group were above the cut-off value and were significantly higher (P = < 0.0001) than unvaccinated pullets (Fig. 1a). During lay, mean antibody titres in the vaccinated group remained above the cut-off value and were significantly higher over the course of the experiment than titres observed for unvaccinated birds (mean log_10_ antibody titre = 2.8) (Fig. 1b).

During rearing, six litter samples from the vaccinated group (two at each time point from shed A) were tested positive by PCR for wild type *Salmonella* spp. following enrichment in BPW. Multiplex PCR results indicated that these were wild type *Salmonellae*. These samples were, however, culture negative. STM-1 was detected in three out of 16 litter samples up to 13 weeks of age (one week after 3rd vaccination).

### Effects of STM-1 vaccination on hens housed in naturally contaminated production farms

Both production farms were positive for wild type *S.* Typhimurium phage type 9. Out of 30 cages sampled at week 17, 10 cages from Farm A (*S*. Typhimurium phage type 9 = 8 cages, *S*. Infantis and *S.* Orion = 1 cage each) and 10 cages from Farm B (*S*. Typhimurium PT 9 = 4 cages, *S*. Infantis, *S*. Agona and *S*. Oranienburg = 2 cage each) were selected for the longitudinal study.

**Fig. 2:**
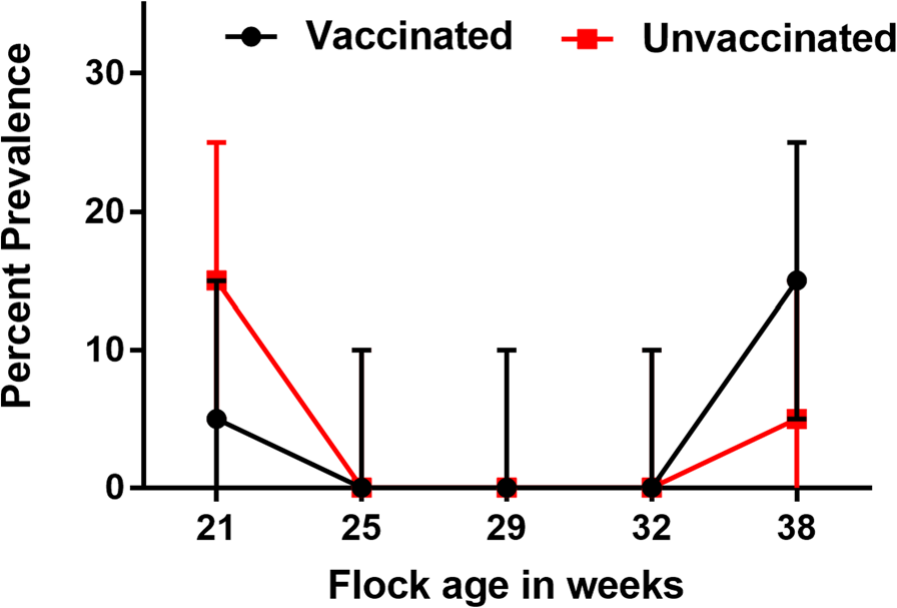
Prevalence (%) of *Salmonella* Typhimurium in faecal samples during early lay in production shed, at each sampling point, fresh faecal samples (n = 10 each from vaccinated and unvaccinated groups) from Farm A and B were collected. Binomial Exact 95% confidence intervals are reported

During early lay no significant difference was detected in the prevalence of *Salmonella* in faeces, as detected by culture. Similarly, multiplex PCR and serotyping results indicated that there was no significant difference in the prevalence of *S.* Typhimurium in the faeces of the vaccinated and unvaccinated groups. *S.* Typhimurium prevalence was higher in faecal samples by the culture method at week 21 and at week 32 (Fig. 2).

**Fig. 3:**
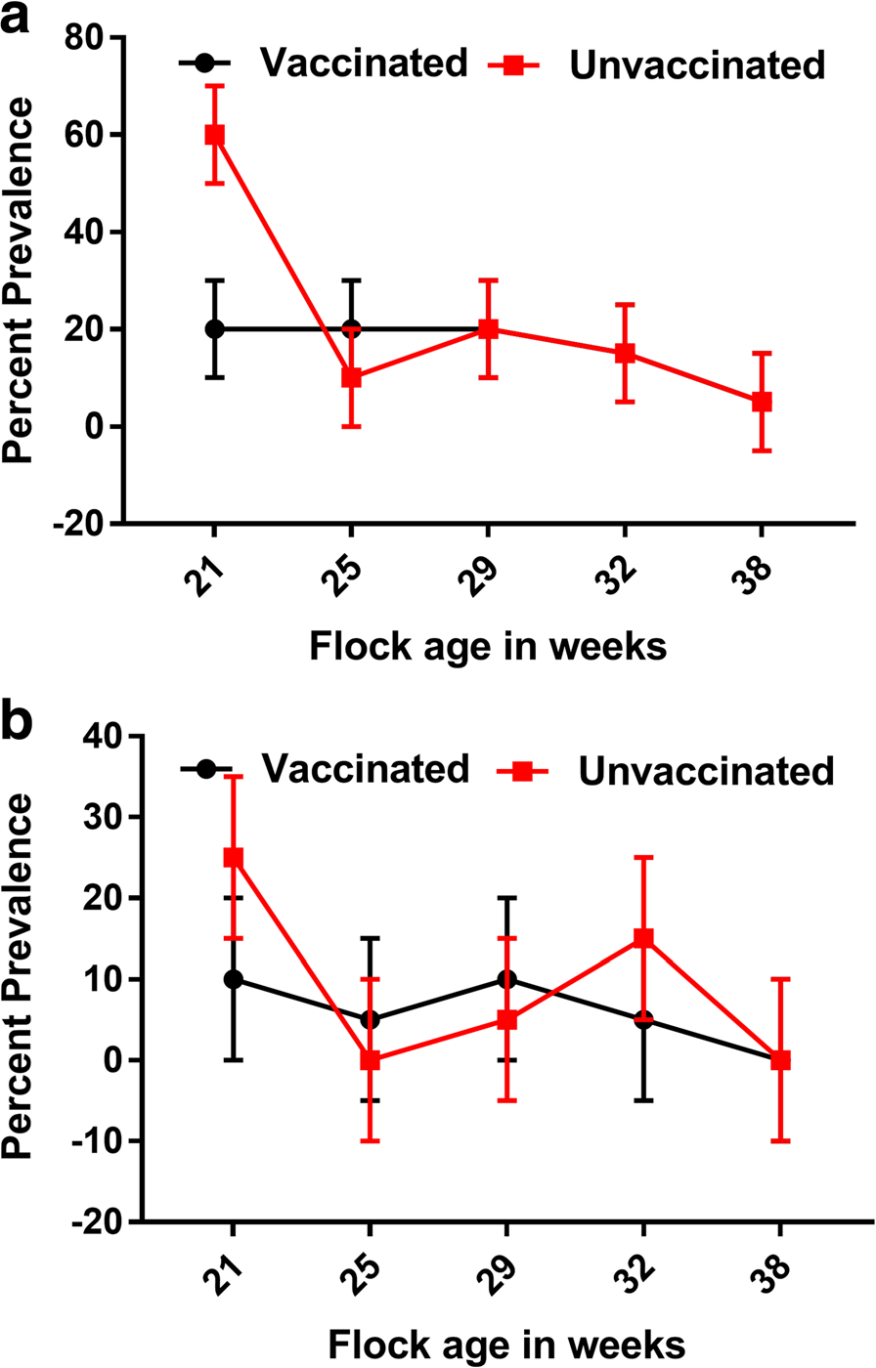
**a** Prevalence of *Salmonella* spp. on egg belt during early lay. **b** Prevalence of *Salmonella* Typhimurium on egg belt during early lay. At each sampling point, Egg belt swabs from cage front (n = 10 each from vaccinated and unvaccinated groups) from Farm A and B were collected. Binomial Exact 95% confidence intervals are reported

**Fig. 4:**
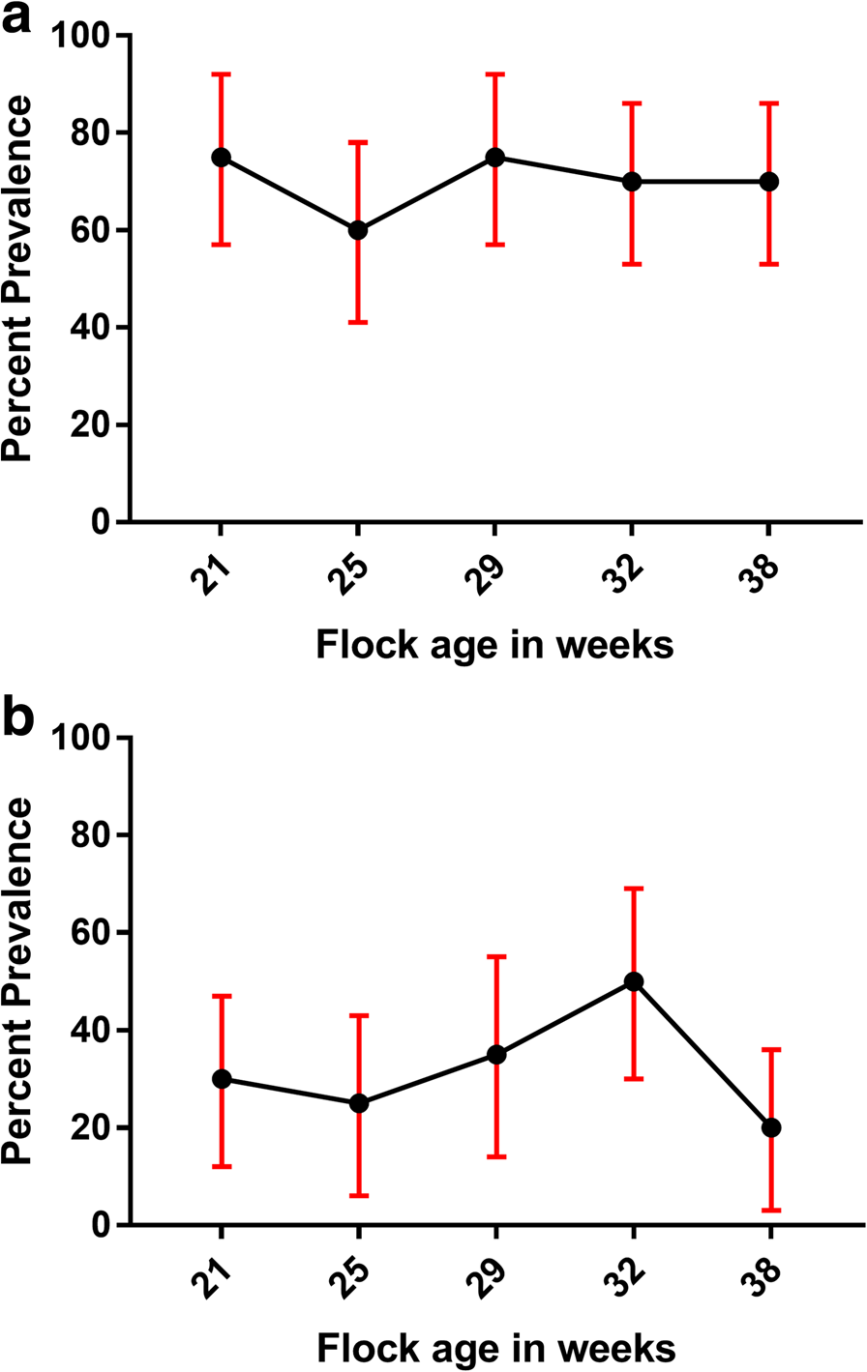
**a** Prevalence of *Salmonella* spp. in dust samples collected at various sampling points during early lay. **b** Prevalence of *Salmonella* Typhimurium in dust samples at various sampling points during early lay. At each sampling point, dust swabs (n = 5 each from vaccinated and unvaccinated groups) were collected from Farm A and B during early lay. Binomial Exact 95% confidence intervals are reported

No significant difference in the prevalence of *Salmonella* or *S.* Typhimurium, as detected by culture, was observed between vaccinated and unvaccinated groups on the egg belt in early lay (Fig. 3a, b). Both wild-type *Salmonella* spp. and *S*. Typhimurium were consistently found, by culture, in dust samples (Fig. 4a, b). Only one eggshell was *S.* Typhimurium positive among samples collected from the vaccinated group. Egg shell samples collected from unvaccinated group were negative for *Salmonella* spp. All egg internal contents were negative by culture for *Salmonella* spp. Four faecal samples were positive for STM-1 by PCR, although STM-1 was not detected by culture. Other serovars detected during this study included *S.* Mbandaka, *S.* Infantis, and *S.* Agona.

**Fig. 5:**
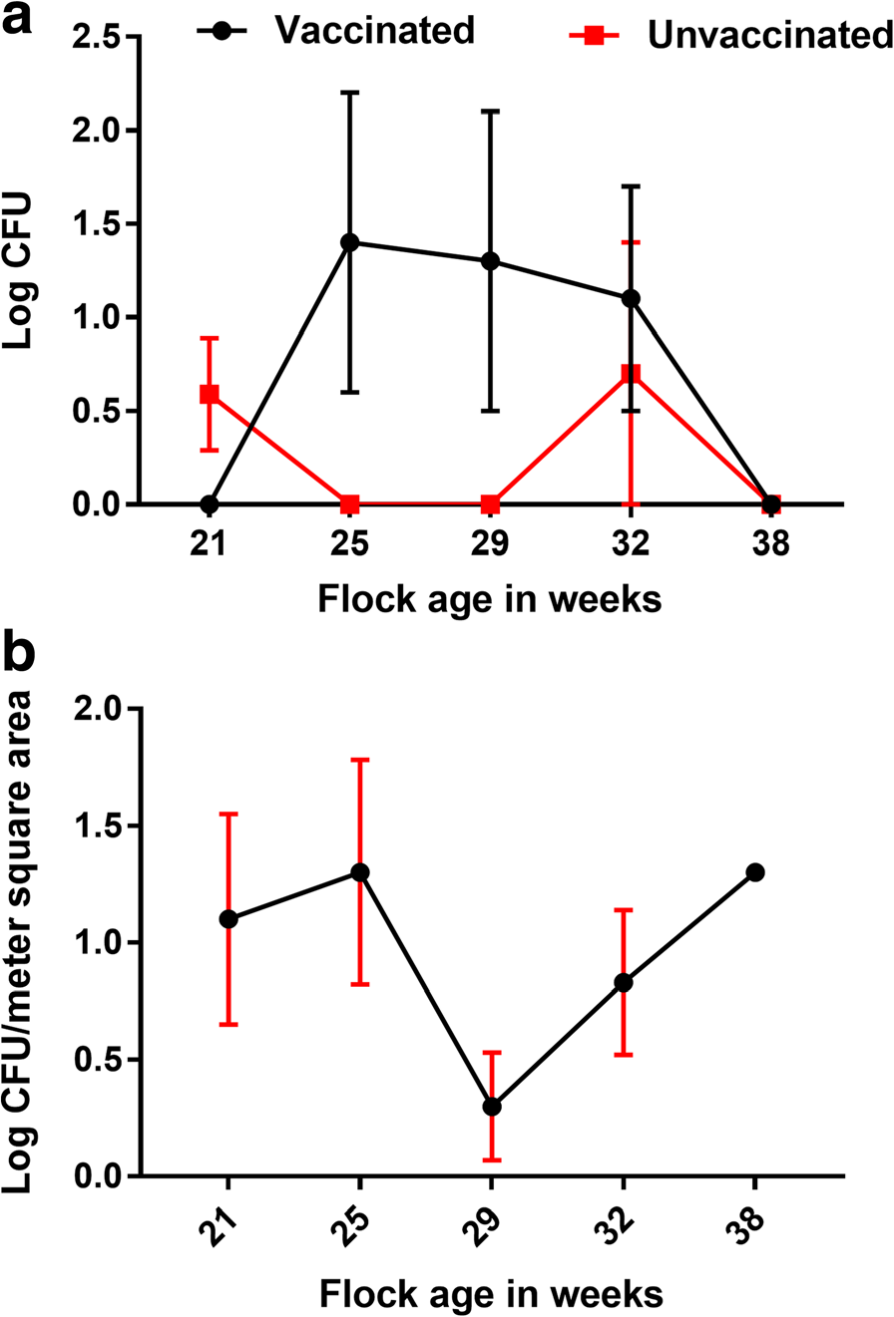
**a** Level of wild type *Salmonella* on egg belt samples from vaccinated and unvaccinated groups over the period of this experiment. (Log CFU ± SE). The bacterial load was measured by qPCR. Data was analysed by ANOVA. There were no significant differences between vaccinated and unvaccinated flocks (**b**) Level of wild type *Salmonella* spp. in the dust collected from shed during the experiment. (Log CFU ± SE). The bacterial load was measured by qPCR. Data was analysed by ANOVA

Egg belt wild type *Salmonella* loads (as detected by qPCR) were not significantly different between treatment groups (Fig. 5a). The level of *Salmonella* spp. in dust samples did not vary significantly over the experimental period (Fig. 5b).

## Discussion

Most vaccines are developed to prevent disease, however there is no evidence that *S.* Typhimurium infection causes disease in adult hens. Thus, the rationale underlying vaccination is to reduce bacterial shedding, and therefore to reduce the environmental and product contamination rates. Two live vaccinations (ocular and oral) followed by parenteral administration (injection) in combination with inactivated oil-emulsion vaccine (EDS + ND) prior to the onset of egg production provides a convenient vehicle for the administration of STM-1 through a multi-dose method minimizing preparation and administration costs. In this trial, *S*. Typhimurium IgG serum antibody titres in unvaccinated birds were below the positive threshold, but the titre was above the threshold in vaccinated hens. This finding was in agreement with a previous trial [14]. Oral *S.* Typhimurium challenge with 10^9^ CFU bacteria is sufficient to produce a strong antibody response in infected hens [15]. Oral administration of Vaxsafe® ST does not induce a strong humoral immune response and only transitory reduction in *Salmonella* colonization of the caeca [14]. Virulent serovars, such as *S.* Typhimurium*,* at high dose are more likely to invade and induce a greater systemic immune response [16]. Based on these observations, it could be concluded that birds in the current trial were not challenged with a high enough bacterial load necessary to induce a systemic immune response.

No significant difference was observed in the prevalence of *S.* Typhimurium in faeces, between vaccinated and unvaccinated groups. Our findings are in agreement with a previous trial [17] reporting that a *S*. Typhimurium *aroA* deletion mutant did not significantly reduce the frequency of faecal shedding of ST under experimental conditions. Another study reported a reduction in faecal shedding of ST in chicks vaccinated with an oral and intramuscular dose of an *aroA* mutant *S*. Typhimurium at four days old [18]. Although this mutant initially reduced the faecal excretion for 14 days post challenge, this effect did not persist. Antibody responses contribute to clearance of extracellular bacteria but *Salmonella* can persist intracellularly, so a cell mediated immune response (CMIR) is essential for clearance [19]. In the present trial, there was an increased antibody response in the vaccinated group after parenteral administration of Vaxsafe® ST although cell-mediated immunity was not evaluated.

Vaccinated birds were dubbed for differentiating vaccinated and unvaccinated flocks. Dubbing can reduce heat transfer from adult bird making these birds vulnerable to heat stress [20]. Stress could induce *S*. Typhimurium shedding in faeces ultimately increasing the risk of egg contamination [21], however, it is important to note, that birds were housed in environmentally controlled sheds. Further work on the effects of STM-1 administration on the CMIR and measurement of stress indicators would contribute to understanding the biology of this vaccine.

The persistence of *S.* Typhimurium in dust samples collected in this study is consistent with a previous report [22]. Environmental samples were positive for ST at all sample intervals. Only four faecal samples and one eggshell were positive for ST. One eggshell positive sample was detected which was not sufficient to assess whether STM-1 had any effect on the shedding of wild type *Salmonella* on eggs. Given that faecal samples, dust and egg belts are indicators of egg contamination [22], it could be deduced that the STM-1 vaccine may not have any effect on egg contamination, although larger controlled investigation is necessary. Eggs were collected directly from the egg belt which was already contaminated with *Salmonella*. Contact with the egg belt, should, therefore have been the primary source of eggshell contamination.

qPCR data revealed that the *Salmonella* load on the sampled commercial farms was low. It is therefore likely that the birds received a small challenge during the trial. During previous epidemiological investigations we were able to detect more than 4 log_10_ CFU in dust samples [22] indicating that the level of *Salmonella* spp. could be variable between different flocks housed in the same shed.

For this trial, the recruitment of farms was largely based on the willingness of farmers to participate. Recruitment of a larger number of egg farms would have been ideal; however, cooperation from egg producers over a period of several months through mid to late egg production period and the requirement of resources are a limiting factor to such studies [18].

## Conclusion

Live vaccines may not be very effective in multi-age sheds because older *Salmonella* infected birds in the shed may serve as a continuous source of bacteria for newly arrived pullets. Further studies are required to investigate efficacy of STM-1 in a single aged commercial flock housed in a shed. Use of STM-1 in multi-age flocks is “not an ultimate intervention” for reduction of *S.* Typhimurium because of the complexities involved in achieving control, such as the efficacy of cleaning of sheds, the lack of resting periods between batches, and possible carry over of contamination from existing flocks. Administration of live vaccine is only one intervention strategy and, if combined with an effective biosecurity program, it may assist in reducing the risk of product contamination.

## Methods

### Pullet rearing farm

A commercial pullet rearing farm with a history of *S.* Livingstone was selected for this study. The farm had three sheds (A, B and C). Shed C housed 15,000 birds. Sheds A and B accommodated 5000 birds each. Surface dust swabs (*n* = 8) and clean wood shavings (n = 8) from a 1 m^2^ area were collected from each shed before resting period and prior to chick placement. Litter samples were collected from the front, middle, and rear sections of each shed. Dust swabs were collected from extraction fans and sidewalls. Sample numbers were determined based on previous findings [23].

### Chick placement, vaccination, and rearing farm

**Fig. 6:**
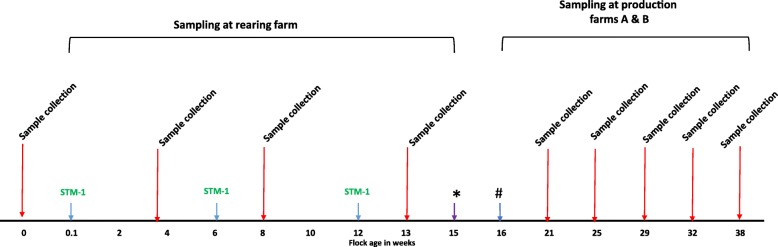
Flow diagram of experimental plan. STM-1 was administered by spray at 0.1 week followed by drinking water at week 6 and then intramuscular at week 12. Red arrows indicate sample collection points during rear and production. Birds were reared on rearing farm up to week 15 and transferred at the point of lay to production farm at week 16. * = Testing of production shed prior to placement at point of lay pullets; # = Placement at point of lay pullets on Farm A and Farm B

Meconium samples (pooled from 90 chicks) were collected from day old chicks at the hatchery. Chicks were randomly divided into two groups. Vaccinated birds (*n* = 10,000) received first dose of Vaxsafe® ST (Bioproperties Pty Ltd., Australia) by coarse spray and their combs dubbed for identification. All other chicks (*n* = 15,000) were left unvaccinated. Vaccinated and unvaccinated chicks were placed in separate boxes and transported to the rearing farm. Vaccinated chicks were placed in sheds A and B while unvaccinated chicks were placed in shed C. At 6 weeks, birds in sheds A and B received second dose of Vaxsafe® ST vaccine (Bioproperties Pty Ltd., Australia) in drinking water followed by third dose by IM injection at 12 weeks (Fig. 6). All together vaccinated birds received three doses of of Vaxsafe® ST vaccine vaccine. The vaccine was reconstituted using a commercial Marek’s disease vaccine diluent under veterinary supervision and administered as a 0.5 mL dose along with a commercial multi-valent Egg drop syndrome (EDS) / Newcastle disease (ND) killed vaccine (Nobilis® EDS + ND, MSD Animal Health). All birds were reared in deep-litter (softwood shavings), floor-based sheds. Antibiotic-free feed was sourced commercially and provided ad libitum. Stocking density at 16 weeks of age was 30 kg/per m^2^. All birds had access to nipple drinker lines. After chick placement, rearing sheds were sampled at 4 weeks, 8 weeks and 13 weeks old. At each time point, 31 composite litter samples and dust swabs were collected from both groups (sheds A = 8, B = 8 and C = 15) in sterile Whirl-Pak plastic bags (Thermo Fisher Scientific, Australia) and processed for *Salmonella* isolation. For the collection of dust swabs, Whirl-Pak speci-sponge bags (Thermo Fisher Scientific, Australia) were pre-moistened with 20 mL of buffered peptone water (BPW; Oxoid, Australia).

### Commercial egg farm sampling

Although the rearing farm had capacity to house 25,000 birds, only two commercial caged egg farms, Farm A (7680 birds) and Farm B (8568 birds) were selected for this study. Farms were recruited for this trial based on geographical proximity, a history of *S*. Typhimurium infection, and participation willingness of producers. Farm A and Farm B had multi-aged flocks in sheds with each age-class housed in separate rows. Prior to placement of study flocks, Farm A already had flocks aged 46, 58 and 64 weeks, all housed in the same shed and Farm B already had a single 64-week old flock in the shed. Cages and sheds were dry cleaned prior to stocking of vaccinated or unvaccinated birds in the shed. On Farm A, the study flock included 1280 cages (6 birds per cage), totalling 7680 birds (5000 vaccinated + 2680 unvaccinated) housed in approximately 1700m^2^ shed. On Farm B, study flock included 1428 cages (6 birds per cage) totalling 8568 birds (5000 vaccinated + 3568 unvaccinated) housed in approximately 1800m^2^ shed.

**Fig. 7:**
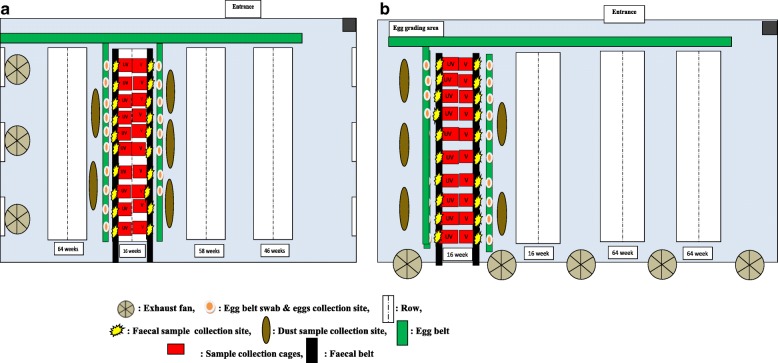
**a** Layout and sampling design of Production farm A sampled during the study. Ten cages per treatment group were selected at 16 weeks of age. Vaccinated birds (5000) + unvaccinated birds (2680). Unvaccinated: UV; Vaccinated: V. **b** Layout and sampling design of Production farm B sampled during the study. Ten cages per treatment group were selected at 16 weeks of age. Vaccinated birds (5000) + unvaccinated birds (3568). Unvaccinated: UV; Vaccinated: V

*Salmonella* infection status of the farms was surveyed by collecting dust and cage swabs (*n* = 8 each) from both sheds one week prior to the arrival of point-of-lay pullets. Vaccinated and unvaccinated pullets were transported at 16 weeks of age from the rearing farm to production farms on the same vehicle. Birds were housed in the same shed on each farm to provide a constant challenge for both treatment groups and to permit potential horizontal transfer of STM-1 to unvaccinated birds (Fig. 7a, b). One week after the arrival of pullets, faecal samples from 30 cages from vaccinated and unvaccinated groups were randomly selected throughout the shed. Ten *Salmonella* positive cages were then selected for longitudinal sampling. Vaccinated and unvaccinated flocks were then sampled at approximately monthly intervals from 21 weeks. Based on previous findings, *Salmonella* shedding is most prevalent in the lower three cage tiers [24], thus cages were selected at equal intervals along the three lowest tiers of the five tiers. Fresh faecal samples (*n* = 10 each from vaccinated and unvaccinated groups) were collected from the manure belt underneath the cages. Faecal samples (*n* = 10), egg belt swabs (*n* = 10), eggs (*n* = 60) and dust swabs (1 m^2^ area) (*n* = 5) were collected during each sampling.

### Collection and processing of environmental samples

All samples were processed for *Salmonella* and STM-1 isolation by culture as described previously [22, 24]. Purple colonies on Brilliance *Salmonella* agar (BSA, Oxoid Australia) were presumed to be *Salmonella*. STM-1 (white colonies) is easily differentiated from wild type *Salmonella* (white colonies with black centres) as it does not produce H_2_S on xylose lysine deoxycholate agar (Oxoid, Australia), permitting presumptive identification. In addition, STM-1 does not grow on BSA [25]. STM-1 or wild type *Salmonella* colonies were added to 0.5 mL of brain heart infusion broth (BHI, Oxoid, Australia) and incubated overnight at 37 °C and then stored in 80% glycerol. Egg belt swabs and dust samples were moistened with 20 mL BPW and processed for *Salmonella* isolation. Presumptive *Salmonella* isolates were sent to the *Salmonella* Reference Laboratory (Adelaide, Australia) for serotyping.

At each sampling, eggs were collected directly from the egg belt in front of each cage into a sterile plastic bag. Six eggs were pooled for processing. Eggshell wash and internal contents were processed separately as described previously [22].

### Serology

During rearing, 30 blood samples (*n* = 15 from each treatment group) were collected in lithium heparin tubes (BD Vacutainer**®** Plus plastic tube, UK) at each sampling. During lay, ten blood samples from each treatment group from each farm were collected. Plasma samples were stored in aliquots and frozen at − 20 **°**C. Antibody titres were determined using an inactivated group B LPS ELISA kit (BioChek, Holland). Titres were calculated according to manufacturer recommendation.

### DNA extraction from faecal, egg belt and dust samples

DNA was extracted from litter, faecal, egg belt and dust samples using the Isolate Faecal DNA Kit (Bioline, Australia) according to the manufacturer’s instructions. The DNA yield (ng) and purity were determined using the NanoDrop® ND-1000 (Biolab, Australia). Dilutions were prepared using nuclease free water to achieve a working concentration of 5 ng/μL DNA.

### Primers for the detection of STM-1 and wild type *Salmonella*

All faecal DNA samples were screened for the amplification of *invA* and *TSR3* genes of *S.* Typhimurium using multiplex PCR as described previously [22]. To differentiate between wild-type and STM-1, primers (Forward 5’-3’GTTTTAAGTGTAATTCGGGG; Reverse 5′-3’ TATGATCAAATGGTTTCGCC) were designed to the transposon / *aroA* gene junction unique to STM-1. This generated an amplicon of 164 base pairs. If a sample was positive for all three PCR products, it was considered STM-1 positive.

To differentiate and quantify wild-type *Salmonella,* primers were designed (forward: 5’-TCTTTTTTCATCCCCACG-3′; reverse: 5’-CGGTTTTACCACAAGCTAA-3′) for the region including the *aroA* gene junction which is conserved for *Salmonella* strains. The specificity of these primers was tested against 22 different *Salmonella* serovars. All serovars except *S*. Mbandaka generated a 182 bp amplicon (Additional file 1: Table S1). No amplicon was observed for samples containing STM-1. This primer set was also used for qPCR.

### qPCR for wild type *Salmonella*

A wild-type *Salmonella* specific qPCR was designed to quantify the amount of bacteria in faecal samples. The total reaction volume was 10 μL and contained 2 μL of sample DNA, 5 μL 2 x Quantifast SYBR Green Master Mix, and 1 μM of both forward and reverse primers. The qPCR was performed with the Quantifast® SYBR® Green qPCR kit (Qiagen, Australia) as per the manufacturer’s instructions. Serial ten-fold dilutions of ST spiked faecal samples were used to generate a standard curve and determine the limit of detection (≥100 of *Salmonella* cells/g of faeces). Negative and positive controls were included in every PCR. The ATCC *Salmonella* Typhimurium strain 14,028 was included as a positive control. Negative control was nuclease free water. Primers designed for detecting wild type *Salmonella* did not amplify STM-1.

### Statistical analysis

Data were analysed using IBM®SPSS Statistics® version 24.0 and GraphPad Prism version 6. STM-1 prevalence was determined using Fisher’s exact test. Bacterial loads and serum antibody titres were analysed using a two-way analysis of variance (ANOVA) followed by a Tukey’s multiple comparison test of the mean. *P* values < 0.05 were considered significant.

## Additional file

Additional file 1: **Table S1.** List of *Salmonella* serovars tested for the specificity of wild type *Salmonella* Typhimurium PCR. (DOCX 15 kb)

## Abbreviations

BHI: Brain heart infusion broth
BPW: Buffered peptone water
BSA: Brilliance *Salmonella* agar
EDS: Egg drop syndrome
ND: Newcastle disease
qPCR: Quantitative PCR
ST: *Salmonella* Typhimurium
