# Supplementary material for: Shedding of Salmonella Typhimurium in vaccinated and unvaccinated hens during early lay in field conditions: a randomised controlled trial
Source: BMC Microbiol. 2018 Jul 20;18:78. doi: 10.1186/s12866-018-1201-0 (PMC6053799; doi:10.1186/s12866-018-1201-0)
Supplement: Supplementary file 1 — Table S1. List of Salmonella serovars tested for the specificity of wild type Salmonella Typhimurium PCR. (DOCX 15 kb) [file 12866_2018_1201_MOESM1_ESM.docx]

Table S1: *Salmonella* serovars and other samples tested against newly designed wild type ST by PCR.

| ***Salmonella* isolate details** | **Result** | ***Salmonella* isolate details** | **Result** |
| --- | --- | --- | --- |
| Typhimurium DT 170/108* | + | Zanzibar | + |
| Typhimurium DT193 | + | Kiambu | + |
| Typhimurium DT135 | + | Virchow | + |
| Typhimurium DT 5 | + | Cerro | + |
| Adelaide | + | Lille | + |
| Orion | + | Ohio | + |
| Agona | + | Bredeney | + |
| Johannesburg | + | Havana | + |
| Livingstone | + | Senftenberg | + |
| subsp I ser 4,12:d:- | + | Oranienburg | + |
| Chester | + | Montevideo | + |
| Mbandaka | - |  |  |

*- Same phage type referred as 170/108.
